# Measuring Calves' Usage of Multiple Environmental Enrichment Objects Provided Simultaneously

**DOI:** 10.3389/fvets.2021.698681

**Published:** 2021-10-01

**Authors:** Ana C. Strappini, Gustavo Monti, Pilar Sepúlveda-Varas, Inès de Freslon, José M. Peralta

**Affiliations:** ^1^Animal Science Institute, Universidad Austral de Chile, Valdivia, Chile; ^2^Preventive Veterinary Medicine Institute, Universidad Austral de Chile, Valdivia, Chile; ^3^Veterinary Clinical Sciences Institute, Universidad Austral de Chile, Valdivia, Chile; ^4^Animal Care and Use Committee, Universidad Austral de Chile, Valdivia, Chile; ^5^College of Veterinary Medicine, Western University of Health Sciences, Pomona, CA, United States

**Keywords:** environmental enrichment, tactile stimulus, calves, object usage, behavior

## Abstract

This study aims to assess calf usage of five potential enrichment devices provided simultaneously. We used 25 weaned Holstein-Friesian calves housed in groups of five (five replicates), and their behavior was recorded continuously with video cameras. This longitudinal observational study used a pen equipped with a mechanical and fixed brush, cowhide, and horizontal and vertical ropes. Data collected included how many visits each object received per day, the type of object usage, and the duration of the visits. Calves used all five objects at least once, and they used items more during the daytime than at night. Brushes were used mainly for grooming (e.g., rubbing or scratching), while ropes and cowhide for oral interactions (e.g., licking, chewing, and biting), most likely to lack oral stimulations that would naturally be satisfied by suckling and grazing at this age. The objects most frequently used were the mechanical brush and the horizontal rope, and they received the highest number of visits (214.9 and 154.9 bouts/day, respectively). The least chosen object was the stationary brush, which had the lowest number of visits (62.9 bouts/day). The provision of multiple enrichment objects for weaned calves should be considered as they may add complexity and novelty to barren environments.

## Introduction

In modern dairy production systems, calf facilities are designed to cover the basic physiological needs of the animals, give them access to feed and water, and offer protection from extreme environmental conditions and a dry area for resting. Unfortunately, these housing systems often do not consider other needs such as grooming or body care (1, 2), which are essential to enhance the calves' overall well-being beyond a basic level. Moreover, a monotonous barren environment can induce the occurrence of behavioral problems (i.e., abnormal behavior and redirected behavior) (3).

It has been suggested that providing farm animals with enrichment objects can prevent frustration and abnormal behaviors (4), promoting better welfare. Several objects have been described as a viable alternative that might provide sensory and occupational enrichment to young stock (5, 6). Some enrichment objects, such as ropes, were reported to elicit the development of oral interactions (i.e., licking, chewing, and biting) (7). While adult cows of both beef and dairy breeds are not very interested in interacting with ropes, calves were reported to interact orally more often with the ropes, showing interest in this object when placed in their pens (8, 9). Previous studies make the rope available for chewing by leaving it hanging loose from the side of the pen (7). It is unknown whether calves could use a rope tied to the wall for oral interactions or grooming.

Grooming behavior, both self-grooming (self-directed) and allogrooming (social directed), have several biological functions for cattle (10). It contributes to the thermoregulation of the body of the animal, minimizes the levels of parasitism by keeping the body clean (of mud, feces, urine, and insects), promotes social interactions between individuals of a group by decreasing agonistic interactions (11), and helps animals to cope with stressful situations, such as after prolonged periods of restrain in self-locking stanchions (12). In natural conditions, bovines use the trunks of trees, poles, shrubs, or other abrasive surfaces to scratch, rub their body, and keep their skin and hair healthy and clean (13). In calves, maternal grooming naturally removes bacterial load and contributes to maintaining the body hygiene of the young animal (14). However, this does not happen under artificial rearing conditions, where the mother is absent. In this case, calves have to search for alternatives to satisfy their motivation for grooming. The provision of cowhides—leather made from the skin and hair of a cow—in pen as a rubbing object has not yet been investigated in calves.

Brushes, both automatic and fixed, have been described as a viable alternative that provides sensory and occupational enrichment to animals, promoting the expression of natural behaviors (3, 5–7). For example, Toaff-Rosenstein et al. (15) described how healthy heifers used the brush in body region grooming. In dairy cows, it has been observed that the brushes contribute to satisfying the natural need for grooming (rubbing and scratching), especially in places that are difficult to reach by the cow, and are associated with a cleaner body (12). In addition, brushes could be tools to monitor the health of animals because their use decreases when animals are sick [e.g., metritis (16), mastitis (17), and health in calves (18)], or to help determine whether they are under conditions of heat stress (19). In terms of production, the physical enrichment with brushes would increase the daily milk production. Schukken and Young (17) found that the difference in milk production was stabilized at 1 kg more in those cows that had used the brush when compared to those that had had no access. Dairy cows are highly motivated to use a mechanical brush. In a motivation test, McConnachie et al. (13) found that dairy cows were similarly motivated to access a mechanical brush and to access fresh feed (TMR). It is unknown if calves use the mechanical brush when there are multiple options for grooming, such as stationary brushes, available in pen.

Knowing what animals like to use provides us with valuable information to make decisions about housing facilities, type of floor, and environmental enrichment items, among others. Understanding calf usage can be a valuable resource when designing facilities and enrichment programs to improve animal welfare in this group of animals. This study aimed to assess calf usage (frequency, duration, and bouts) of different enrichment objects when provided together. We achieved this objective in weaned calves by investigating the use of the objects with unlimited access to them for 7 days. Additionally, we assessed factors (time of the day and individual or social use) associated with each object's frequency and duration of use.

## Materials and Methods

### Place of the Study and Study Design

This study was carried out at the Austral Agricultural Experimental Station of the Austral University of Chile, located in Valdivia, Chile (39°46′42 S, 73°13′38 W). The data collection began in September 30th and ended in November 14th 2019.

A longitudinal study on 25 female weaned Holstein–Friesian calves was used, with an average age of 86 days (SD ± 6.8) and an average weight of 72.8 kg (SD ± 10.4). During the pre-weaning period, calves ingested the first colostrum from the mother, and on the day of birth, they were housed into individual pens where they received 4 L/day of colostrum. Subsequently, they started with milk replacer 4 L/day, twice a day. After 10 days, calves were moved to the group pen, where they took 5 L/day in the automatic feeder (average intake 1.3 L at a time). Calves were weaned at 75 days of age. Regarding the solid diet, calves started with 150 g/day of concentrate and hay on demand. The concentrate was gradually increased to 1.5 kg/day at 75 days of age. Weaning was carried out gradually, decreasing milk intake from week 8 to week 10. Then, the provision of concentrate increased to 2 kg/day, and hay was provided on demand. During post-weaning period, calves were housed in group pens of 10 individuals at the calf unit of the experimental station. During the post-weaning period, calves were housed in group pens of 10 individuals at the calf unit of the experimental station. For the study, the animals were divided into five groups of five calves (one furnished pen × five repetitions) balanced for age and weight. Then, the calves were moved to the test area about 100 m from the calf unit, where they remained 8 days in the pen and then were moved back to their calf unit facilities.

The procedures of this study were approved by the Animal Care Ethics Committee of the Universidad Austral de Chile (Committee Approval N° C45-2020).

### Animals, Housing, and Management

**Figure 1:**
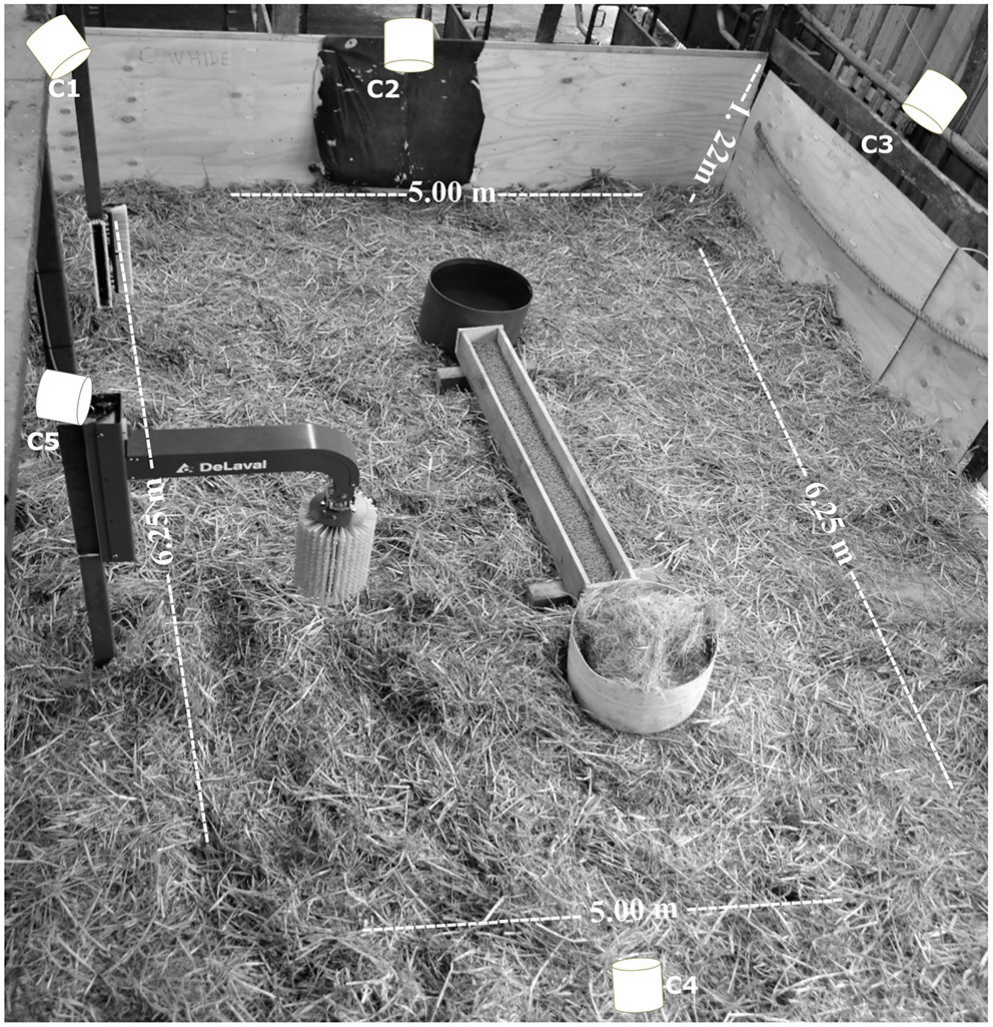
Pen layout, with pen size and cameras location (C1–C5).

Before the onset of the study, each calf was weighed and identified with a large unique number painted with a non-toxic marker (Donaghys, Dairy Mark, NZ) on each flank. Next, calves were housed in groups of five at a space allowance of 4.5 m^2^/calf, in a wooden enclosed pen with straw bedding (Figure 1). Hay and concentrate (2 kg/calf/day) were provided daily (between 8:00 and 9:00 a.m.), and calves had *ad libitum* access to water. When the test ended, the bedding material was removed, the empty pen was cleaned and disinfected, and fresh straw was added before a new group of five calves entered the test pen for the subsequent replication.

### Study Design

**Figure 2:**
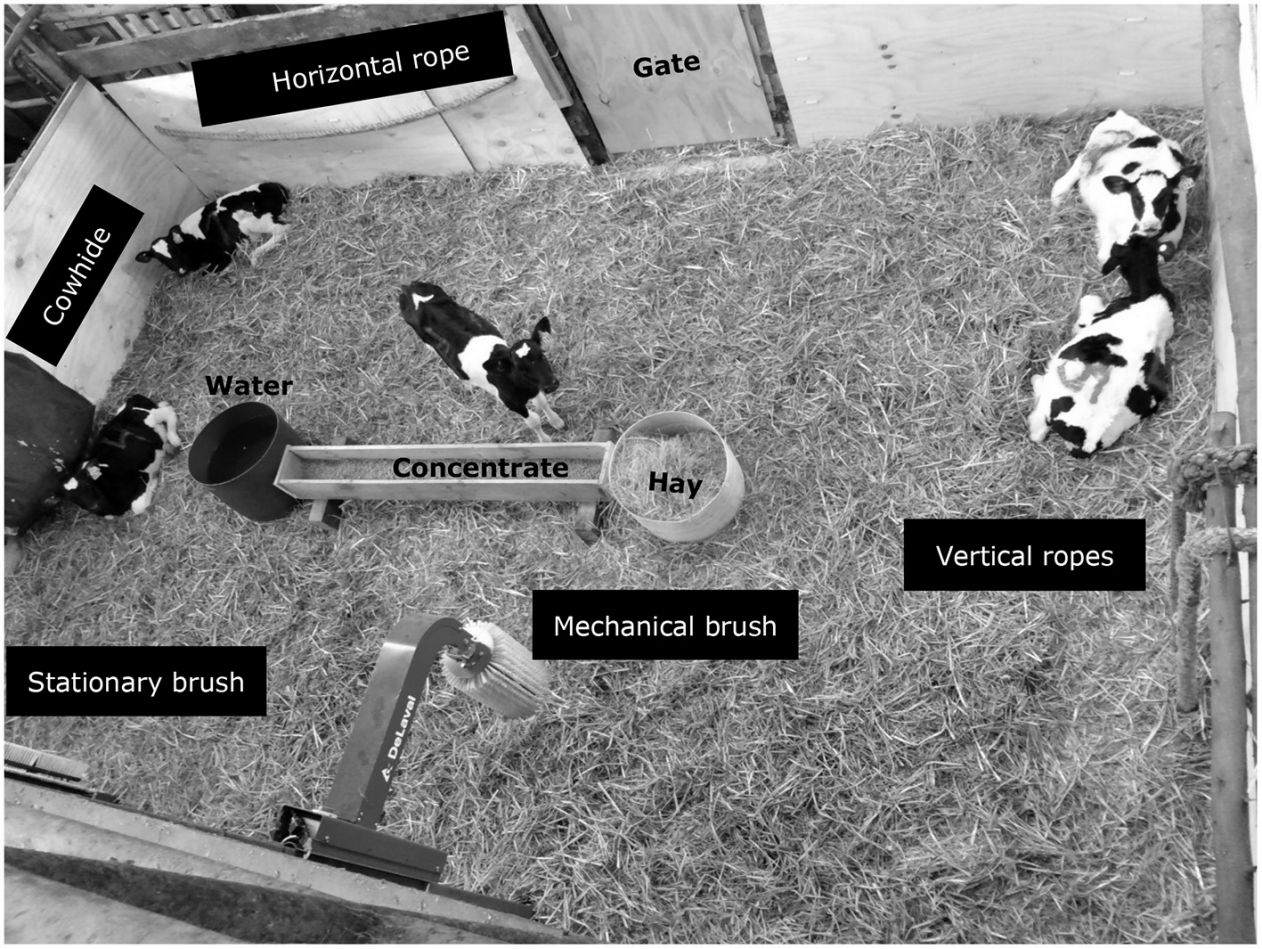
Pen layout with location of the enrichment objects, hay, concentrate feeders, and water container.

A longitudinal observational study was carried out in a furnished pen (Figure 2). Calves' behaviors were described for five types of enrichment objects. These objects were as follows:

- A mechanical brush (mini swinging brush, MSB, DeLaval, Sweden) with nylon bristles and a sensor that initiated a gentle speed (25.5 rpm) rotation movement when an animal moved it (72 cm length × 13 cm width × 30 cm height).
- Two small identical fixed brushes (65 cm length × 7 cm width) with thick nylon bristles arranged vertically on a metallic column.
- A commercial cowhide with skin and hair of a cow filled with sawdust.
- A horizontal rope, made from natural manila fibers (2.50 m long, 2.0″ thick); and
- A vertical rope made from natural manila fibers (3.50 m long, 2.0″ thick) was arranged in double parallel lines.

Enrichment devices were securely mounted into walls or structures in the pen and were placed at a height over ground level that was easily accessible to the calves' face, neck, and trunk, so the calves could freely use them any time and at will. Brushes, ropes, and hide could provide physical and tactile enrichment to the young calves. All calves were naïve to brushes, ropes, or hides.

The study comprises eight consecutive days, where the first day was considered the day of acclimation (d0) for socially adjusting calves to each other, and its data was excluded from the analysis.

The study was replicated five times using the same facilities, conditions, objects, and object location in pen. The daily frequency of object use, the type of use (individual or social), and the duration were described.

### Behavioral Measurements

The behavior of the calves was continuously recorded with five infrared video cameras (Ezviz CS-CV310-A0-1B2WFR, Hangzhou, China) mounted 3 m above the pen. During the 7 days of assessment (d1…d7), the following behaviors were recorded (Table 1):

- Use of enrichment object (individual or group),
- Rubbing, and
- Manipulating an object with the mouth.

**Table 1:** Ethogram designed to describe the use and type of object usage with five enrichment objects offered simultaneously.

**Behavioral category**	**Behavior**	**Description**
Individual	Mechanical brush use	A calf is physically in contact (head, neck, back, or rump) with the brush for more than 5 s; calf can be still or moving its body in an up-down or side-to-side motion or licking, nibbling, or biting the brush.
	Stationary brush use	A calf is rubbing its body moving back and forth against the brush; calf is licking, nibbling, or biting the brush.
	Cowhide use	A calf is moving its body in an up-down or side-to-side motion while in contact with the hide; calf is licking, nibbling, or biting the hide.
	Horizontal rope use	A calf is moving its body in a side-to-side motion while in contact with the horizontal rope; calf is licking, nibbling, or biting the rope.
	Vertical rope use	A calf is moving its body in an up-down or side-to-side motion while in contact with the vertical rope; calf is licking, nibbling, or biting the rope.
Group	Use object with others	More than one calf are simultaneously in physical contact with the same object (rubbing, licking, nibbling, or biting).

The object usage was classified as individual when a single calf was in contact with the item and social when the event involved two or more calves.

The first replicate was used to determine the frequency of calf activities during day and night. The grooming behavior and oral manipulation of objects by five calves allocated to the first study group was recorded 24 h a day for seven consecutive days (168 h/calf) to evaluate whether day and night object use was different in nature and frequency. The *day* was defined as the time of the day between sunrise and sunset (from 6:00 a.m. to 6:00 p.m.), and *night* as the time between sunset and sunrise (from 6:01 p.m. to 5:59 a.m.) (12-h day/12-h night).

One trained researcher analyzed the behavioral data. Each behavior, start and end times, was recorded using the Behavioral Observation Research Interactive Software (BORIS) (20), which logged times with 1/100° s accuracy.

### Health Assessment

Before entering the experimental pen, calves were inspected by a veterinarian to identify clinical signs of disease, injuries, and abnormalities. Any calf that presented one of these conditions was excluded from the study and replaced by a healthy one. For those included in the study, during the time it lasted, the same veterinarian evaluated each calf's health status daily, using a modified version of the Calf Health Scoring System of the University of Wisconsin (available at https://www.vetmed.wisc.edu/fapm/wp-content/uploads/2020/01/calf_respiratory_scoring_chart.pdf).

Any calf suspected of being ill during the experiment was examined, isolated from the group, received the appropriate pharmacological treatment, and excluded from the study. Therefore, only clinically healthy animals participated in the study. Nevertheless, during the study period, none of the calves included got sick or had to be replaced.

### Statistical Analysis

All the data were analyzed using R statistical software (V.9.3) (21) using calf as the experimental unit of analysis. Significant differences were declared at *P-*value. Descriptive statistics and bout characteristics were summarized by object, behavior, individual calf, time, and day of the study. First, the general daily use was described for each object in terms of total duration and frequency; then, the behavior durations and frequencies were summed for each calf and for a 12-h observation period.

A *bout* was defined as a specific behavior sequence (Table 1) lasting more than 5 s. *Total daily bout duration* was defined as the sum of all bouts' duration performing the behavior (min/day), and the *average bout duration* was calculated as total bout time divided by bout frequency (bout/day).

A generalized linear mixed model (or GLMMs) was used to assess which variables could be associated with the frequency of using objects. They are an extension of linear mixed models that allow response variables from different distributions, such as count responses. The Poisson regression was used to analyze both count data and rate data and determine which explanatory variables (*X* values) affect a given response variable (*Y* value, the count or a rate). The model analyzed the bout counts under the assumption that all populations have the same trend, but at different levels (due to differences between individuals, day of study, and between replicas of the study), corresponding to a random-intercepts model.

However, given that bout counts of several individuals were measured on the same individuals over time, the assumption of independence of measurements within individuals was no longer suitable. Therefore, instead of using a conventional Poisson model, we used a Poisson mixed-effects regression model (22), accounting for it. It included a subject-specific random effect introduced in the *linear predictor* to seize the dependence, and other random effects included in the model were the day of study and replicates.

To assess different objects' use during the day or nighttime, we run a preliminary analysis using a model that included as fixed-effects the items (brushes, ropes, and cowhide) and the moment of the day. Next, the model included as fixed-effects the items (brushes, ropes, and cowhide), behavior (manipulate an object with the mouth or rubbing), the moment of the day (morning or afternoon), social use (individual or group), age, and weight at starting day. Data exploration was carried out first to look for outliers. Then, the conditional model was constructed using a forward approach; main effects and interactions were assessed, and it used the Akaike information criterion (AIC) and the Bayesian information criterion (BIC) indexes for evaluation of model goodness-of-the-fit. Finally, collinearity was assessed by using variance inflation factor (VIF) and a correlation matrix.

Specific *post hoc* pairwise comparisons were made between the brush and other items using contrast statements in R library emmeans (23).

A generalized linear mixed model was used to analyze factors associated with the duration of rubbing and manipulation with the mouthing behavior, considering the same variables as the previous model, to account for repeated measures and replication of the study. The overall variability was decomposed by incorporating random effect terms to account for within-cluster homogeneity in outcomes due to individual, replica, and day of study. For all regression model calculations, we used the lme4 package (24) of R (21).

## Results

### Preliminary Analysis: Use of the Objects During Day and Night

**Figure 3:**
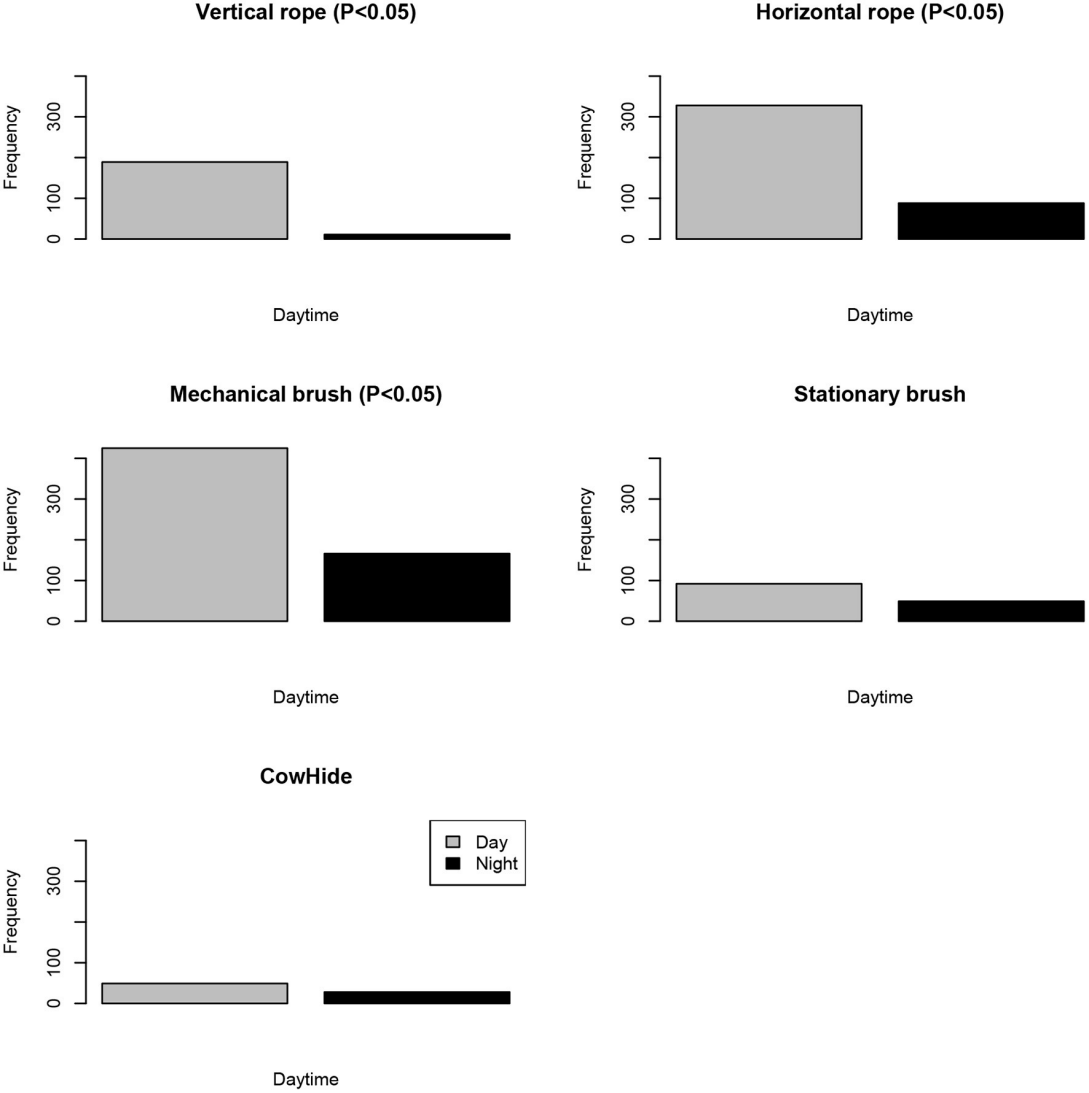
Bout frequency of use (total number of bouts use/per time of the day) of five enrichment objects recorded continuously by day according to time of day (day/night) from preliminary analysis using five calves. Statistically significant differences between times of day are indicated (*P* < 0.05).

We recorded 1,199 total bouts of object use displayed by five weaned calves during day and night for seven consecutive days. The enrichment objects were used with higher frequency during the daytime hours than night hours (76 vs. 24%, respectively; *P* < 0.001, Figure 3). However, at night, the number of visits to the five items decreased significantly compared to the number of visits at daytime (290 vs. 909, respectively; *P* < 0.001).

Calves were more active during daytime hours than during nighttime. Therefore, for the rest of the experiment, video-tracking analysis was performed for daytime hours. Next, behavioral data for the other four replicates were retrieved from video-camera files based on a 12-h schedule and analyzed for all five replicates (from 6:00 a.m. to 6:00 p.m.). However, to maintain homogeneity in the analysis, overall results from the study were obtained and presented using daytime records only as follows.

### Use of Multiple Enrichment Objects

#### Frequency of Use of the Objects

All calves (*n* = 25) used the five enrichment objects at least once during the study. Overall, the mechanical brush was the most frequently used (1,504° bouts/week), followed by the horizontal rope (1,084 bouts/week). The stationary brush was the least frequently used object (440° bouts/week).

It was observed that the mechanical brush was used daily by most of the calves of the experiment (average 24 per day, range 22–25 calves per day), followed by the horizontal rope (22 calves per day), considering the total number of calves used in the five replicates (25 calves). Further, the vertical rope and the cowhide were visited by a smaller number of animals (20 calves per day for each object). Interestingly, the daily number of animals that used the different items decreased across study days, except for the mechanical brush that was used evenly throughout the study.

Most of the items received the highest number of visits during the first day of the study. After that, the frequency of use changed over time, with visits decreasing from day 1 to day 6 for most objects. The exception was the cowhide, which did not receive as much attention during the first 5 days, and then its use peaked on day 6. In general, it seems that decreased novelty over time reduced the usage of the brushes and ropes across the study, although they remained in use throughout (Table 2). In general, we observed that for all objects, except for the cowhide, the use of enrichment objects decreased from day 4 onwards, and after that, the use fluctuates day by day, but these differences were statistically not significant.

**Table 2:** Average (mean ± SD) number of visits to the enrichment objects per day of the study, for using five enrichment objects observed in 25 calves kept in groups of five for five replicates recorded continuously 12 h per day, for 1 week, based on raw data.

**Object**	**Day of the study**						
	**1**	**2**	**3**	**4**	**5**	**6**	**7**
Vertical rope	2.7 (2.6)	1.7 (1.0)	1.7 (1.0)	1.2 (0.5)	1.3 (0.6)	1.5 (0.7)	1.5 (0.9)
Horizontal rope	3.5 (3.0)	2.4 (1.8)	2.2 (1.2)	1.8 (1.3)	2.2 (1.8)	1.9 (1.3)	2.6 (1.6)
Mechanical brush	3.8 (3.4)	2.6 (2.7)	2.9 (2.4)	2.5 (2.0)	2.9 (2.4)	2.3 (1.8)	2.9 (2.4)
Stationary brush	1.7 (1.2)	1.4 (0.9)	1.3 (0.6)	1.5 (0.6)	1.2 (0.6)	1.4 (0.7)	1.3 (0.6)
Cowhide	2.0 (1.4)	1.6 (1.1)	2.2 (1.6)	1.5 (1.1)	1.8 (1.1)	3.3 (2.9)	2.1 (1.7)

Overall, group use of the objects occurred at a lower rate than in solitary (15.1 vs. 84.9%). The horizontal rope was the object that stimulated more social use (26.2%), followed by the cowhide and the vertical rope. Both stationary and mechanical brushes were mainly used individually (95.5 and 92.4%, respectively). Calves used the mechanical brush individually mainly for rubbing their head and neck (71.6%) and less frequently the back (1.9%) and rump (0.9%). Simultaneously, the use of the mechanical brush by two or more calves at once was rarely observed (7.6%).

The final model (Table 3) shows that the mechanical brush was the most frequently used object followed by the horizontal rope; however, there is no statistically significant difference between both objects (0.24 vs. 0.14; *P* > 0.05). In contrast, cowhide (0.81), vertical rope (0.66), and stationary brush (0.49) were less frequently used (*P* < 0.05). The stationary brush was the least frequently used object by the calves. The comparison between all other categories indicated that differences in use frequencies between pairs of objects were statistically significant, except for the pair horizontal rope vs. mechanical brush and horizontal rope vs. stationary brush as mentioned before. In addition, the model showed that behaviors like rubbing increase the frequency compared to oral manipulation. Finally, calves in a group (two or more) use the objects simultaneously less frequently in comparison with the individual user, and the difference was statistically significant (*P* < 0.0001) (Table 3).

**Table 3:** Final conditional mixed Poisson model for factors associated with the frequency of object usage (*n* = 4,195 s bouts).

**Variable**	**Categories**	**Estimate**	**95% CI**	***P***
Intercept		2.70	2.20; 3.29	0.09
Object	Mechanical brush	Ref.		
	Stationary brush	0.49	0.44; 0.56	<0.0001
	Horizontal rope	1.00	0.89; 1.12	0.98
	Vertical rope	0.66	0.59; 0.75	0.0001
	Cowhide	0.81	0.72; 0.91	0.0006
Behavior	Manipulate object with mouth	Ref.		
	Rubbing	1.10	1.00; 1.21	0.052
Social use	Individual	Ref.		
	Group	0.63	0.58; 0.69	<0.0001

*AIC = 5,717.0; BIC = 5,771.0*.

There was variation among individual calves (*n* = 25), day of the study (1–7), and replicates (*n* = 5). After decomposing the overall variability, 27.7% could be attributed to differences between animals, 39.9% to the day of study, and 32.4% to replications.

#### Duration of Use of the Objects

The distribution of the objects' duration of use was analyzed, and records with a duration longer than the 95th percentile of the distribution were considered outliers and removed from further analysis.

**Figure 4:**
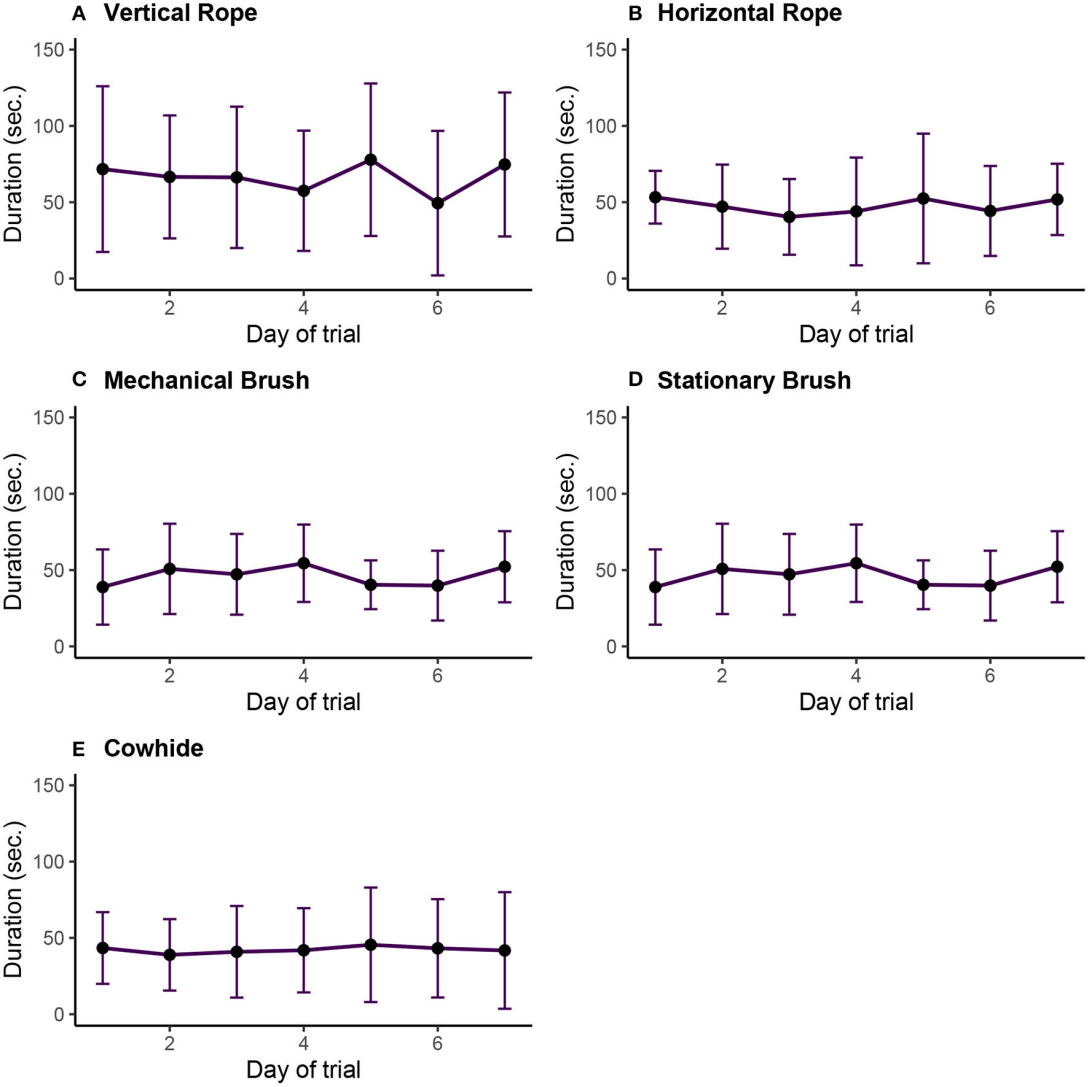
**(A–E)** Average (mean ± SE) of time spent interacting with each environmental enrichment object by day of study for using five enrichment objects observed in 25 calves kept in groups of five for five replicates recorded continuously 12 h per day, for 1 week, based on raw data. **(A)** Vertical rope, **(B)** horizontal rope, **(C)** mechanical brush, **(D)** stationary brush, and **(E)** cowhide.

Overall, calves spent more time interacting with the mechanical brush and the ropes (both horizontal and vertical) (Figure 4) than the stationary brush and the cowhide.

Table 4 summarizes the final conditional model for factors associated with the short or long duration of the bouts. There are statistically significant differences in the duration of object use. The time that calves spent using each object depended on the type of object; however, there were no statistically significant differences by the moment of the day. The duration of the use of the stationary brush was shorter (14.3 s) (Table 5) than the use of the mechanical brush. In contrast, the use of the vertical rope was statistically significantly longer (9.6 s). The duration in the use of the cowhide and the horizontal rope was not statistically different when compared with the use of the mechanical brush (*P* > 0.05) (Table 4). The comparison between all other categories indicated that the differences in duration between pairs of objects were statistically significant, except for the pair cowhide vs. horizontal rope, cowhide vs. vertical rope, and horizontal rope vs. vertical rope.

**Table 4:** Final conditional mixed generalized linear model for factors associated with the duration of the bout use (*n* = 4,195 s bouts).

**Variable**	**Category**	**Estimate**	**95% CI**	***P***
Intercept		57.4	47.2; 67.7	<0.00001
Object	Mechanical brush	Ref.		
	Stationary brush	−14.3	−20.3; −8.2	0.00016
	Horizontal rope	0.8	−3.8; 5.3	0.96
	Vertical rope	9.6	4.0; 15.2	0.0003
	Cowhide	−4.2	−9.9; 1.5	0.21
Moment of day	Morning	Ref.		
	Afternoon	−3.1	−6.6; 0.4	0.09

*AIC = 36,753.2; BIC = 36,808.5*.

**Table 5:** Daily use (mean ± SD), daily bout frequency (mean ± SD), and duration (mean ± SD) for using five enrichment objects observed in 25 calves kept in groups of five for five replicates recorded continuously 12 h per day for 1 week.

**Object**	**Daily use (min/day)**	**Daily bout frequency (mean ± SD)**	**Bout duration (s/bout)**
	**(mean ± SD)**		**(mean ± SD)**
Vertical rope	14.2 (2.4)	112.0 (42.8)	67.1 (47.4)
Horizontal rope	22.8 (7.4)	216.8 (88.0)	47.6 (29.9)
Mechanical brush	29.7 (11.5)	300.8 (129.5)	46.4 (25.1)
Stationary brush	7.4 (2.2)	88.0 (22.6)	38.7 (35.1)
Cowhide	13.7 (14.0)	121.4 (79.7)	42.2 (30.6)

Finally, the model indicates that events that occurred during the afternoon lasted 3.1 s less than those occurring in the morning, but the difference was not statistically significant (*P* = 0.09) (Table 4). There were variations among individual calves and replicates; however, the contribution to overall variability was much smaller than for frequency. After decomposing the overall variability, only 6.2% could be attributed to differences between animals, and 13.6% could be attributed to the variability between replications. The variability between days of study was not significant and, consequently, it was removed from the model.

## Discussion

The present study presents evidence of how weaned calves use different enrichment objects when they are provided simultaneously.

The use of enrichment objects increased during daylight hours compared with night use (76 vs. 24%). This finding could be explained by the circadian rhythm of bovine behavior [reviewed by Kilgour (25)], showing how cattle are generally less active and tend to rest more at night. A daily rhythm of calf activity has been described and shows a low activity level from 8:00 p.m. to 07:00 a.m. (18). Once the daily pattern of activities was established, the rest of the replications consisted of observations conducted only during the daytime.

The enrichment items were always available and accessible to the calves in pen. All calves used each of the objects provided at least once during the study. This finding is in accordance with previous studies that reported calves using a great variety of devices when they were available in pen (4, 8). It was the case for automated brushes (6, 14, 18), stationary brushes (6, 26), ropes (9, 13), hay (27), hanging balls (28), rubber chains, and “calf lollies” (PVC pipes capped on both ends filled with dry molasses) (26). All these examples evidence that young calves are curious and have a high motivation to explore their environment when they have the opportunity (28). Exploratory behavior allows animals to have a comprehensive map of their surroundings and, therefore, to be able to master it (29). In our study, calves had the freedom to explore their environment, and this gave them opportunities to freely choose how to spend their time using the different enrichment objects they had at their disposal.

The interest in the use of most objects decreased over time, showing that habituation to the static enrichment objects can occur rapidly after 3 days for the vertical rope or after 5 days for the stationary brush and the horizontal rope. It is consistent with previous studies that have demonstrated that calves, especially those not raised in isolation, learn to recognize novel objects in their environment and then habituate to their presence (30). The exception was the mechanical brush. The motivation of the calves to use this device remained consistent throughout the experiment, suggesting that the use of rotating mechanical brushes might be sustained by the need for grooming or by the fact that they change from a static to a dynamic state when in use and that may make them more attractive. Our results are in line with Velasquez-Muñoz et al. (18), who reported that brush use was stable across time in heifer and bull calves, observed from week 4 (pre-weaning) until week 7 (weaning). Moreover, we can speculate as Kohari et al. (31) observed that calves incorporated elements of play behavior when they used movable rubbing or scratching objects, explaining the higher motivation for visiting the automated brush.

Weaned calves in the present study used brushes mainly for grooming (rubbing/scratching), while ropes and cowhide were used for oral manipulations (licking, nibbling, and biting). Therefore, our findings do not indicate which enrichments are most important for calves since they were used for different functions. Calves spent more time manipulating objects with their mouth than rubbing or scratching their body. This latter use is likely related to the young age of the recently weaned calves that retain a strong motivation for suckling or might be for chewing grass. Similar results were reported by Kohari et al. (14), who provided pre-weaned pair-housed calves with an automated brush and a hanging rope, finding similar differences in how the animals used the enrichment substrates. Ropes—both horizontal and vertical—were used more in groups of calves than brushes; this might be because these objects offer a larger contact area for more than one calf at a time. It seems that access to environmental devices might be beneficial for the socialization and welfare of cattle (26, 32). Bulens et al. (26) investigated the effect of the environmental devices on the social behavior of beef calves. They found that calves housed in enriched pens (with cattle brush) displayed significantly (*P* < 0.0001) more play and social behavior than calves in non-enriched pens. Future research could record social facilitation concerning rope use, the number of individuals involved and the type of social interaction, and whether they are agonistic or affiliative. While ropes promoted licking and chewing behavior, the mechanical brush allowed grooming, which helps cattle satisfy the need to engage in this natural behavior (13, 33). Multiple enrichment items can satisfy different types of needs, further promoting the calves' welfare.

This observational study allowed us to estimate the frequency of using of multiple novel objects when they are offered together. The most frequently used objects were the mechanical brush and the horizontal rope, which were visited the most and by the most significant number of animals during the 7 days of testing.

A previous study (34) showed that adult dairy cows are highly motivated to interact with automatic brushes. This fact was observed in mangy cows (with cutaneous acariasis), even after being treated for the ectoparasite when they are no longer pruritic (15). Our results show that weaned calves used brushes for grooming, spending ~30 min a day using the mechanical brush, most of the time alone. There were clear periods of high mechanical brush usage during the day, with peaks early in the morning, after feeding time, midday, and at the end of the afternoon (data not shown). A recent study (35) characterized the diurnal activity of weaned beef calves concerning the use of a brush, finding that calves used the device mainly during daylight hours, as was seen in the present study. Calves used the mechanical brush mainly for grooming their head and neck (71.6% of the visits). It was expected since previous studies in cattle reported that interactions with the brush were focused on the head and neck (36, 37). According to Leruste et al. (38), cattle scratch themselves on inanimate objects to reach inaccessible parts of the body, such as the head, neck, back, and hindquarters, which is also consistent with our findings.

The horizontal rope was most frequently used for oral behavior (licking, biting, or chewing), maybe because suckling is still a relevant behavioral need for recently weaned calves. In addition, these non-nutritive oral activities might be linked to the lack of suckling associated with limited milk availability (39). As the calves in our study were already weaned, it could be hypothesized that they were chewing on the ropes because they were hungry; however, there were traces of concentrate in the feed-trough for much of the observation time, and hay was freely available. These non-nutritive oral activities are multi-factorial behaviors, but they are generally considered redirected to engage in a particular behavior that cannot be satisfied in the environment (7, 38, 39). In our study, the lack of access to fresh forage may also influence the occurrence of non-nutritive oral behaviors such as chewing, licking, and biting the ropes.

Surprisingly, the least used object was the stationary brush, which recorded the lowest percentage of visits. The lack of interest in this item may have been affected by its closeness to the mechanical brush in the experimental pen (Figure 1). The complexity of the automatic brush could have distracted the calves' interest away from the stationary model. The rotating movement of the mechanical brush started when a calf pushed it and continued to rotate for 10 s until the brush remained vertical (38). Zobel et al. (7) suggested that this type of brush has a continued visual effect on calves even after usage. The mechanical brush is more “flexible,” allowing calves to reach more areas of their body, and does not require specific brush movements. In contrast, the fixed brush was static with no active interaction component for the calves, which may have chosen not to engage as often. It would be worthwhile to determine the frequency of use of the fixed brush by calves when there is no mechanical brush present in the pen or when they are located further apart, as there are reports that indicate that the use of stationary brushes decreases the incidence of unwanted redirected behaviors, like cross-suckling (6).

However, it is essential to note that the duration of use for different objects with different functions cannot be compared, so each object's importance in terms of welfare cannot be concluded based on these results alone.

## Conclusions

We observed a clear diurnal pattern used for all enrichment items, consistent with the normal circadian rhythm of activity and rest that characterizes dairy cattle. Mechanical brush and horizontally placed rope were the most commonly used enrichment objects for weaned calves in terms of frequency, duration, and the daily number of animals using the substrates.

In calf-rearing systems, the provision of multiple enrichment items should be considered to improve their welfare in monotonous environments.

## Data Availability Statement

The original contributions presented in the study are included in the article/Supplementary Material, further inquiries can be directed to the corresponding authors.

## Ethics Statement

The animal study was reviewed and approved by Animal Care Ethics Committee of the Universidad Austral de Chile (Committee Approval N° C45-2020).

## Author Contributions

AS was responsible for the conception of the study, study design, manuscript writing, and revisions. IF was responsible for the acquisition of data and manuscript revision. PS-V was responsible for study design, health evaluation of calves, and revision of the manuscript. GM was responsible for the statistical analysis, data analysis, and revision of the manuscript. JP was responsible for the conception of study, data interpretation, and manuscript editing. All authors contributed to the article and approved the submitted version.

## Funding

This study was part of the project UACh-De Laval N° 10110200, the use of a mechanical brush to enrich the environment of artificially reared calves and its effects on health and welfare. We acknowledge DeLaval S.A., Chile, for the financial support.

## Conflict of Interest

The authors declare that the research was conducted in the absence of any commercial or financial relationships that could be construed as a potential conflict of interest.

## Publisher's Note

All claims expressed in this article are solely those of the authors and do not necessarily represent those of their affiliated organizations, or those of the publisher, the editors and the reviewers. Any product that may be evaluated in this article, or claim that may be made by its manufacturer, is not guaranteed or endorsed by the publisher.
